# Improving delirium care in the intensive care unit: The design of a pragmatic study

**DOI:** 10.1186/1745-6215-12-139

**Published:** 2011-06-06

**Authors:** Noll L Campbell, Babar A Khan, Mark Farber, Tiffany Campbell, Anthony J Perkins, Siu L Hui, Greg Abernathy, John Buckley, Regg Sing, Jason Tricker, Mohammad Zawahiri, Malaz A Boustani

**Affiliations:** 1 Department of Pharmacy Practice, Purdue University School of Pharmacy, West Lafayette, IN, USA; 2 Indiana University Center for Aging Research, Indianapolis, IN, USA; 3 Regenstrief Institute, Inc., Indianapolis, IN, USA; 4 Wishard Health Services, Indianapolis, IN, USA; 5 Department of Medicine, Indiana University School of Medicine, Indianapolis, IN, USA; 6 Richard L. Roudebush Veterans Administration Medical Center, Indianapolis, IN, USA; 7 Indiana University Health, Indianapolis, IN, USA

## Abstract

**Background:**

Delirium prevalence in the intensive care unit (ICU) is high. Numerous psychotropic agents are used to manage delirium in the ICU with limited data regarding their efficacy or harms.

**Methods/Design:**

This is a randomized controlled trial of 428 patients aged 18 and older suffering from delirium and admitted to the ICU of Wishard Memorial Hospital in Indianapolis. Subjects assigned to the intervention group will receive a multicomponent pharmacological management protocol for delirium (PMD) and those assigned to the control group will receive no change in their usual ICU care. The primary outcomes of the trial are (1) delirium severity as measured by the Delirium Rating Scale revised-98 (DRS-R-98) and (2) delirium duration as determined by the Confusion Assessment Method for the ICU (CAM-ICU). The PMD protocol targets the three neurotransmitter systems thought to be compromised in delirious patients: dopamine, acetylcholine, and gamma-aminobutyric acid. The PMD protocol will target the reduction of anticholinergic medications and benzodiazepines, and introduce a low-dose of haloperidol at 0.5-1 mg for 7 days. The protocol will be delivered by a combination of computer (artificial intelligence) and pharmacist (human intelligence) decision support system to increase adherence to the PMD protocol.

**Discussion:**

The proposed study will evaluate the content and the delivery process of a multicomponent pharmacological management program for delirium in the ICU.

**Trial Registration:**

ClinicalTrials.gov: NCT00842608

## Background

In 2005, approximately 2.7 million Americans aged 65 and older spent at least one day in the intensive care unit (ICU) costing Medicare an estimated $27.5 billion [1-3]. It is estimated that up to 80% of these older ICU patients had delirium during their hospital stay [4]. Older adults with delirium are more prone to falls, injuries, pressure ulcers, restraints, mortality, institutionalization, and dementia [4-8]. Delirium is considered an acute brain failure that is most often unrecognized among older adults in the ICU [9-14]. These patients may receive potentially harmful medications such as anticholinergics and benzodiazepines that are thought to cause or worsen delirium [15-18].

We conducted a systematic evidence review of the existing literature to evaluate the efficacy and safety of various pharmacological interventions [16]. Of the four studies focusing on delirium treatment, only one study suggested a benefit of haloperidol and chlorpromazine in reducing delirium severity, though no differences in delirium incidence, duration, or hospital length of stay were identified [19].

Despite the lack of evidence, clinicians that care for these patients often use typical and atypical antipsychotics, benzodiazepines, and other sedatives to manage the symptoms of delirium [20,21]. This approach conflicts with our current understanding of the neurotransmitter balance in delirium. The available literature suggests a therapeutic role for acetylcholine enhancement, gamma-aminobutyric acid (GABA) reduction, and dopamine reduction [9-12,15,22], which is reflected in available clinical practice guidelines for delirium management [4]. Therefore, this neurotransmitter model supports a combination intervention that includes avoiding or reducing the use of benzodiazepines and anticholinergics, and the use of low dose antipsychotics such as haloperidol [4,23-26]. However, there have been no randomized controlled trials evaluating the efficacy of this approach on reducing delirium severity, duration, or complications [15-18].

Our primary objective is to test the efficacy of a pharmacological intervention in reducing delirium severity and duration among adults cared for in the ICU. Our primary hypothesis is that, in comparison to usual care, intervention patients will have (1) reduced delirium severity at one week following randomization or hospital discharge, and (2) fewer hospital days with delirium or coma. The secondary hypothesis theorizes that the study intervention will result in (1) shorter hospital lengths of stay, (2) lower ICU, hospital, and 30-day mortality, and (3) lower hospital-acquired complications related to delirium.

## Methods/Design

### Ethical Approval

The study has been approved by the Institutional Review Board (IRB) of Indiana University Purdue University Indianapolis. Approval for enrollment in the study requires informed consent provided by the potential participant's legally authorized representative.

### Study Population

Wishard Memorial Hospital (WMH) serves as Indiana's only public acute care hospital for the indigent population with 340 staffed beds, of which 51 are intensive care unit beds. The ICU at WMH includes a medical ICU (MICU), surgical ICU (SICU), coronary ICU (CCU), and progressive ICU (PICU). In 2008, WMH provided 88,502 patient days of care throughout all hospital beds. The general population demographics of patients admitted to WMH include 59% African-Americans and 68% females [27]. Demographics of patients admitted to critical care units at WMH are included in Table 1.

**Table 1:** Demographic summary of WMH ICU population

Variable	SICU	MICU + CCU	PICU
**Number of beds**	8	14	29

**Average monthly admission**	40	67	228

**Female**	35%	38%	42%

**Age**			
- **< 60**	72%	71%	70%
- **61-80**	27%	25%	26%
- **> 80**	1%	4%	4%

**Admitted from**			
- **ER**	55%	61%	52%
- **Non-ICU wards**	20%	19%	12%
- **Other**	1%	20%	36%

**Mean length of ICU stay, days**	5.96	6.10	3.72

**Discharge location**			
- **Home or other facility**	12%	14%	38%
- **Regular hospital wards**	29%	22%	51%
- **PICU**	47%	50%	--
- **Other**	12%	12%	11%

**ICU mortality rate**	11%	12%	1.3%

*SICU = Surgical intensive care unit; MICU = Medical intensive care unit; CCU = Coronary care unit; PICU = Progressive intensive care unit; ER = Emergency room

### Enrollment and Eligibility

Patients admitted to the ICU for at least 24 hours will be eligible for screening for the study. Screening measures, further described below, will include a measure of the level of sedation using the Richmond Agitation-Sedation Scale (RASS) [28] and the Confusion Assessment Method for the ICU (CAM-ICU) to evaluate for delirium [29]. Criteria for inclusion in the study consist of: (1) patients admitted to any WMH ICU ward (SICU, PICU, MICU, CCU) for ≥ 24 hours, (2) aged ≥ 18 years, (3) score positive for delirium based on the RASS and CAM-ICU on any day during the ICU stay, and (4) are English-speaking. Patients are excluded for a history of severe mental illness, admission with an alcohol-related delirium, admission for an aphasic stroke, have a history of allergic reaction or contraindication to haloperidol, are pregnant or nursing, or have previously been enrolled in the study.

**Figure 1:**
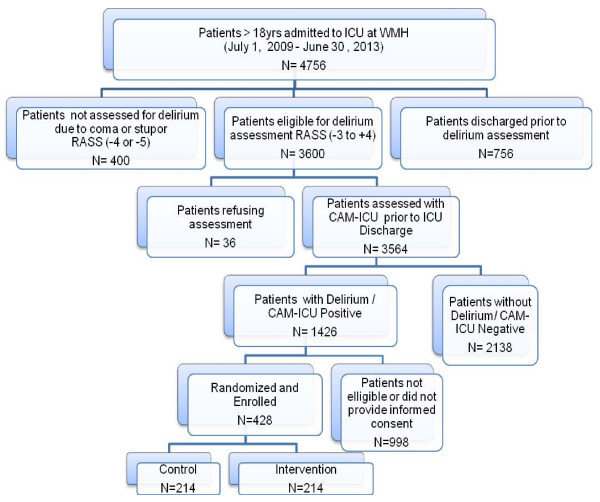
**Expected PMD recruitment flow chart**. ICU: Intensive Care Unit; WMH: Wishard Memorial Hospital; RASS: Riker Agitation Sedation Scale; CAM-ICU: Confusion Assessment Method for the Intensive Care Unit

Patients meeting inclusion criteria will be pursued for informed consent by contacting a legally authorized representative. Figure 1 describes the flow of patients through the screening and enrollment plans of the study. As seen in Figure 1, we anticipate approaching approximately 4700 patients for screening in order to enroll 428 in the randomized controlled trial.

### Design and Delivery of the Intervention

In 2005, we utilized the information technology infrastructure at WMH to develop a patient-specific computerized decision support system (CDSS). This system targeted hospitalized older adults with cognitive impairment (CI), including those with delirium. This study was called enhancing care for hospitalized older adults with memory problems (e-CHAMP). The intervention was designed to: (1) alert the clinician of the presence of CI in his/her patient at the time of ordering a diagnostic or therapeutic order; (2) identify a medication order with a moderate to severe central anticholinergic activity and suggest an alternative medication; (3) identify the presence of bladder catheterization and suggest an early discontinuation; and (4) identify a physical restraint order and suggest pharmacological alternatives using a low dose of haloperidol [30].

This prior clinical trial experience revealed valuable lessons that translated to the design of the current CDSS in the intensive care setting. First, many alerts were not routinely updated, and often presented conflicting or confusing recommendations that were outdated. Second, the end-users were bombarded with numerous computer-generated alerts and reminders during their routine care of hospitalized older adults [31,32]. The reputation for the clinical relevance of these computer-based alerts, warnings, and reminders was poor amongst clinicians. Furthermore, the end-users developed what was referred to as the "F8" or "escape" button reaction, where the end-user automatically ignored the alerts. Thus, we know that computer-based decision support may be a useful resource, but by itself cannot change provider behavior in our institution.

In response to the e-CHAMP experience, we elected to support the computerized delivery of the study intervention with human intelligence in designing the current trial, the Pharmacologic Management of Delirium (PMD). Realizing the pace of care in the ICU environment is rapid and changes quickly, the constantly present computerized component described below will be activated for each study patient enrolled in the intervention arm. In addition, a clinical pharmacist with training in cognitive impairment and delirium (NLC) will review medication orders twice daily to carry out the study intervention. The inclusion of a clinical pharmacy specialist will allow for review of medications that may be ordered prior to enrollment in the study as well as identifying medications that may be ordered outside of the capabilities of the CDSS, such as non-formulary drugs. Additionally, the inclusion of a human interaction will improve adherence to study interventions as well as document reasons for protocol deviations.

#### Infrastructure of the Computerized Intervention: The Medical GOPHER Physician Order-Entry System

Since 1984, the Medical GOPHER system, a network of computer workstations, has allowed physician order entry and other direct interactions between computer and physician [33]. The Medical GOPHER system is used by physicians to write all orders at WMH and is linked to the Regenstrief Medical Record System (RMRS). It allows the computer to push information to providers as a CDSS. Furthermore, it can do this at the critical time of clinical decision making, while the physician is initiating an order or recording a diagnosis. The study intervention will utilize the GOPHER system to alert any practitioner entering an order (either original or verbal) for any of twenty medications with definite or strong anticholinergic properties that the patient is enrolled in the study and the medication is not recommended. Additionally, the intervention will provide a list of alternatives to consider depending on the intended use of each particular medication.

#### Regenstrief Medical Record System at WMH

The computerized Regenstrief Medical Record System (RMRS) is the primary instrument for processing data and monitoring patient and physician activity at WMH. RMRS is a modular system, capable of recording appointments, laboratory, pharmacy, and physician ordering activity. This comprehensive database captures all inpatient and outpatient data by date in a fully coded form, making patient care data readily retrievable. Additionally, RMRS contains death certificate information for registered patients who die in, or outside of, Indiana. The RMRS will be used to evaluate secondary study outcomes such as length of stay and mortality.

### Description of the Pharmacologic Intervention

The study intervention targets the imbalance of three neurotransmitters believed to play a role in delirium. Our intervention will focus on the pharmacologic contribution of those neurotransmitter systems through a combination of human and electronic activity. The CDSS will be employed in reducing the role of anticholinergic medications in delirium, whereas the human interaction by the study pharmacist will execute benzodiazepine monitoring, initiate haloperidol as described below, and ensure adherence to anticholinergic reduction recommendations provided by the CDSS.

#### Reduction of anticholinergic medications

Using lists of medications with central anticholinergic activities identified from the medical literature well as values of serum anticholinergic activity (SAA) measurement, our interdisciplinary aging brain team developed the Anticholinergic Cognitive Burden (ACB) scale. The scale is intended to be a practical tool to identify the severity of negative anticholinergic effects on cognition of prescribed and over-the-counter medications. The team established the following scoring system: drugs with possible anticholinergic effects (as demonstrated by the SAA or the in-vitro affinity to muscarinic receptors but with no clinically relevant negative cognitive effects) were given a score of 1. Drugs with established and clinically relevant cognitive anticholinergic effects were given the score of either 2 or 3, based on each drug's blood-brain permeability and its association with the development of delirium identified in available literature [12].

Medications included in the ACB having a score of 2 or 3 will be the targeted medications for anticholinergic burden reduction. Each order for a definite anticholinergic will trigger a warning message to avoid its use in patients with delirium, along with alternative recommendations and prompts to discontinue the offending agent. The definite anticholinergics targeted in our study as well as suggested alternatives are available in Appendix 1.

#### Benzodiazepine reduction

Withdrawal of benzodiazepines in intervention patients will follow recommendations from the Society of Critical Care Medicine (SCCM) guidelines regarding the use of sedative and analgesic medications in critically ill patients [4]. Benzodiazepine reduction will occur cautiously but deliberately, with constant communication with the primary medical or surgical team and with close monitoring for intolerable withdrawal reactions. Benzodiazepine dose reductions will be initiated with 20-40% dose reduction on day one, with subsequent reductions of 10-25% every 24 hours as tolerated. Safety and tolerability of the weaning schedule will be evaluated through routine nursing assessments of vital signs and behavior documented every four hours.

#### Low-Dose Haloperidol course

During the ICU stay, every patient who consents to the study, randomized into the intervention group, and is not under mechanical ventilation will received an order of 0.5 to 1 mg haloperidol every 8 hours via oral or parental route for a total of seven days or until discharge from the hospital. Because of age-related pharmacokinetic differences in metabolism and pharmacodynamic sensitivity, patients under the age of 60 years will be given 1 mg/dose, and those aged 60 years or older will be given 0.5 mg/dose. This low dose of haloperidol is based on the critical analysis of the data from the clinical trials that tested the use of antipsychotics in delirium and the drug metabolism of psychotropics in older adults. This low dose is not intended to replace the sedative regimen used in patients receiving mechanical ventilator.

Intravenous haloperidol use has been associated with a higher risk of cardiovascular adverse outcomes, specifically QT prolongation and Torsades de Pointe. However, in a recent systematic review of case reports of haloperidol-associated arrhythmias, a wide range of haloperidol doses were used prior to the inciting events (2-1540 mg). The onset of most events was typically within minutes to hours following the cumulative doses of haloperidol (range 15 minutes to 5 days). The authors concluded that haloperidol given intravenously appears safe when given in doses less than 2 mg. Patients enrolled in our study will not be given haloperidol if they have: (1) a QT prolongation of 500 milliseconds or above, (2) a history of seizure disorders, or (3) an indication for seizure prophylaxis (i.e., severe stroke or traumatic brain injury) as haloperidol may decrease the seizure threshold [34].

### Usual Care

Those randomized to the usual care group will receive no electronic or human reminders for pharmacologic management of delirium throughout their hospital stay. Study participants in the usual care arm may receive haloperidol as part of routine care in the ICU, without restriction on the dose or frequency administered. Of note, study participants will be randomized at the patient level, therefore providers will care for patients enrolled in both intervention and usual care arms of the study, exposing the risk of cross-contamination. Study personnel will continue to visit the patients twice daily to perform delirium, safety, and adverse event assessments.

### Randomization

Following the eligibility and screening assessments, informed consent will be obtained from the participant's legally authorized representative. Randomization of eligible patients providing consent will occur in a 1:1 ratio between intervention and usual care groups. The computerized order entry system (GOPHER) is capable of performing this process and will conduct randomization in random blocks of four to encourage even distribution of patients between the intervention and usual care groups.

### Evaluation/Assessment Parameters

#### Primary Outcome

*Delirium Recognition: *In accordance with national guidelines [4], the study will identify delirium by using the RASS and the CAM-ICU on all patients who are admitted directly from the emergency room or transferred from other services to the ICU. Such assessment will be performed after 24 hours of ICU admission and twice daily until discharge from the hospital. As an initial step in conducting the CAM-ICU, the interviewer must assess the patient's sedation status to assure a valid CAM-ICU result. The CAM-ICU was validated using the RASS, and therefore this will be the sedation scale utilized for our study evaluation. RASS has excellent inter-rater reliability among adult medical and surgical ICU patients and has excellent validity when compared to a visual analogue scale and other selected sedation scales [28]. The RASS requires 30-60 seconds to perform with minimal training and has been used by our local ICU team. The RASS is a 10-point scale that reflects the patient level of sedation as determined by their responses to verbal versus physical stimulation. A RASS score of -5 indicates a comatose state with lack of response to verbal or physical stimuli, a score of 0 represents alert and calm, and a score of +4 indicates a state of combative, violent behavior with immediate danger to staff, self, or others. A patient with a RASS score of -3 to +4 will be considered eligible to be assessed by the CAM-ICU to determine the presence of delirium [29].

The CAM-ICU will detect delirium among patients receiving ICU care at both the time of ICU admission and during their ICU stay. The CAM-ICU was chosen because of its practical use in the ICU wards, its acceptable psychometric properties, and based on the recommendation of national guidelines [4,35]. The CAM-ICU score is determined by examining the patient for (a) acute and fluctuating changes in mental status, (b) inattention, (c) disorganized or incoherent thinking, and (d) altered level of consciousness. A CAM-ICU score is considered to be positive if the patient displays both a and b, plus c and/or d. The CAM-ICU diagnosis of delirium was validated against the DSM-III-R delirium criteria determined by a psychiatrist and found to have a sensitivity of 97% and a specificity of 92% [36]. The CAM-ICU has been developed, validated and applied into ICU settings and multiple investigators have used the same method to identify patients with delirium [36-38].

*Delirium Severity: *Since the CAM-ICU does not evaluate delirium severity, we selected the Delirium Rating Scale revised-1998 (DRS-R-98) [39,40] developed by Dr. Paula Trzepacz and colleagues. The DRS-R-98 was designed to evaluate the breadth of delirium symptoms for phenomenological studies in addition to measuring symptom severity with high sensitivity and specificity. It has been used in treatment, phenomenological and pathophysiological studies and has been translated into 12 languages. Characteristic symptoms include impairments of attention, short and long-term memory, visuospatial ability and orientation, perceptual and sleep-wake cycle disturbances, abnormalities of language and thought process and content, motor agitation and retardation, and mood lability. The DRS-R-98 is a 16-item clinician-rated scale with anchored items descriptions corresponding to both symptoms and temporal aspects of delirium. The severity scale has 13 items each rated from 0 to 3 where the sum has a maximum of 39 points, with higher scores indicating greater severity of delirium. Three additional items (rated from 0 to either 2 or 3) capture temporal course and attribution to an underlying etiology and when added to the sum of the 13 symptom items produce the DRS-R-98 total score that ranges from 0 to 46. The DRS-R-98 has excellent inter-rater reliability (intra-class correlation 0.97) and internal consistency (Cronbach's alpha 0.94) [39,40].

#### Secondary Outcomes

The study will collect demographic and baseline functional information from the patient's legally authorized representative and/or caregivers. Cognitive function status will be obtained by interviewing the patient's legally authorized representative using the Informant Questionnaire on Cognitive Decline in the Elderly (IQCODE). IQCODE is a questionnaire that can be completed by a relative or other caregiver to determine whether that person has declined in cognitive functioning. The IQCODE lists 26 everyday situations where a person has to use their memory or intelligence. Each situation is rated by the informant for amount of change over the previous 10 years, using a Likert scale ranging from 1-much improved to 5-much worse. The IQCODE has a sensitivity between 69% to 100% and specificity of 80% to 96% for dementia [41].

Utilizing the electronic medical record system (RMRS), we will collect several data points of interest at baseline and throughout the study period. First, RMRS will allow us to collect reason for admission, severity of illness (APACHE II) [42], and the number of comorbid conditions (Charlson Comobidity Index) [43] for each patient enrolled in the study. We will also collect length of stay (both ICU and total hospital stay), mortality rate, and hospital-related consequences. We have previously defined hospital-related consequences to include: the number of patients with documented falls, use of physical restraints, injuries such as pulling out IV lines or urinary catheters, any ordered or observed re-intubation, and pressure ulcers. These will be assessed using the RMRS, direct daily observation, and retrospective review of the electronic medical record. This definition of delirium-related hospital complications has been previously used and published [44,45].

Use of all medications, including focused intervention-related medications (anticholinergics, benzodiazepines, and haloperidol) will be monitored through the GOPHER computerized order entry system, as well as the McKesson medication administration system. The McKesson system is used at the patient's bedside and collects the administration of each medication. Additionally, McKesson is able to monitor medications administered as continuous infusions by documenting start times, stop times, and drip rates throughout the administration period.

In our study an adverse event will be defined as any untoward medical occurrence in a subject without regard to the possibility of a causal relationship. Adverse events will be collected after the subject has provided consent and enrolled in the study. If a subject experiences an adverse event after the informed consent document is signed (entry) but the subject has not started to receive study intervention, the event will be reported as not related to study drug. All adverse events occurring after entry into the study and until hospital discharge will be recorded. An adverse event that meets the criteria for a serious adverse event (SAE) between study enrollment and hospital discharge will be reported to the local IRB as an SAE. If haloperidol is discontinued as a result of an adverse event, study personnel will document the circumstances and data leading to discontinuation of treatment. A serious adverse event for this study is any untoward medical occurrence that is believed by the investigators to be causally related to study-drug and results in any of the following: Life-threatening condition (that is, immediate risk of death); severe or permanent disability, prolonged hospitalization, or a significant hazard as determined by the Data Safety Monitoring Board. Serious adverse events occurring after a subject is discontinued from the study will NOT be reported unless the investigators feels that the event may have been caused by the study drug or a protocol procedure. Investigators will determine relatedness of an event to study drug based on a temporal relationship to the study drug, as well as whether the event is unexpected or unexplained given the subject's clinical course, previous medical conditions, and concomitant medications.

Although haloperidol is widely used in treatment of delirium, it is not approved by the FDA for such use, and its efficacy in such treatment is not clear. Although our study employs a low dose (1.5 mg per day) of haloperidol for a short period of time (seven days), its potential adverse effects of QT prolongation and extrapyramidal symptoms (EPS) will be monitored during the study. The study will monitor for the following movement-related adverse effects daily through patient examination and chart review: dystonia, akathisia, pseudoparkinsonism, akinesia, and neuroleptic malignant syndrome. Study personnel will use the Simpson-Angus [46] and Barnes Akathisia [47] scales to monitor movement-related effects.

### Planned Analysis

#### Data Analysis

We will conduct intention-to-treat analyses to test the intervention effects on the primary outcomes. No interim analysis is planned for early termination due to efficacy. To formally test for potential harm due to the intervention, 3 interim analyses will be performed using the proposed primary analysis methods after the completion of 0.25, 0.5 and 0.75 of the projected 428 evaluable subjects, using the O'Brien-Fleming boundary, for an overall significance level of 0.05 at study completion.

To test the intervention effect on severity of delirium, we will use the primary outcome of DRS-R-98 severity score on day 8 post-randomization. The primary analysis to test the intervention effect on day-8 DRS-R-98 severity score will be analysis-of-covariance (ANCOVA) with the baseline score as a covariate, after testing and removing any intervention-by-baseline score interaction. If there is a significant interaction, we will test the intervention effect at the mean of the baseline DRS-R-98 severity score. We will use the last observed DRS-R-98 forward to Day 8 as the primary outcome in one analysis. For sensitivity analyses, we will use different methods to impute the missing data. For example, we will impute the missing Day 8 DRS-R-98 severity scores with their worst score on record for patients who have been discharged with delirium or died before day 8. For patients who were hospitalized but could not be evaluated for whatever reason on day 8, we could also interpolate their previous and subsequent DRS-R-98 severity scores to estimate the missing Day 8 score.

We will also test the intervention effect on the repeated measures of DRS-R-98 severity scores using a mixed-effects model. An appropriate contrast for the day 8 effect will be used to corroborate the results of the ANCOVA in the primary analyses. This analysis will provide additional information on the time course of the severity of delirium from its onset in the ICU, with or without intervention.

To test the intervention effect on duration of delirium, we will compare between groups the number of coma-free and delirium-free days in the first 8 days post-randomization using a two-sample t-test corroborated with the Wilcoxon rank sum test. The intervention effect will be further tested while controlling for patient characteristics using multiple regression. Patients who die before day 8 will have all subsequent days counted as not coma-free or delirium-free. We will impute all days post-discharge as coma-free and delirium-free.

For secondary outcomes, binary measures, e.g. mortality and complications, logistic regression will be used to test the intervention effect, controlling for covariates when appropriate. Highly skewed outcomes such as hospital and ICU length of stay will be compared between groups using nonparametric tests, such as Wilcoxon's rank sum test, and semiparametric survival analyses which can control for covariates.

#### Data Safety Monitoring Board

A data safety monitoring board (DSMB) consisting of five members having no participation in the planning or execution of the study will be assembled. The DSMB will be responsible for the oversight of the safety of study patients and to ensure an appropriate sample is collected throughout the study period. The board will review relevant safety data after the completion of data collection of 25%, 50%, and 75% of the planned study sample. During these safety analyses, the DSMB will make recommendations to continue or terminate the progress of the study based on the observed effects of the study intervention.

## Discussion

There are limitations within our study that are worth addressing. First, the potential for contamination exists by randomizing study subjects at the patient level. Physicians may care for patients in both groups and therefore may change behavior based on exposure to the study recommendations. Second, we did not design the study to determine which of the three pharmacologic interventions contributed most significantly to the outcome. Based on our previous experience with e-CHAMP and that of other delirium trials that revealed a small effect size, we chose to evaluate only the impact of the combined pharmacologic intervention. Finally, the impact of the study intervention may be limited by the use of a low dose of haloperidol and the absence of an absolute restriction on anticholinergic and benzodiazepine use. The use of a low dose of haloperidol is a result of our interpretation of the available literature on delirium treatment [16].

Despite these limitations, our study will add valuable information to the scant literature about the hospital care for adults with delirium. First, it provides a focused intervention aimed at the current theory of neurotransmitter imbalances thought to occur during delirium. Second, it provides an opportunity to study the impact of a multi-component intervention not only on delirium duration, but also delirium severity, which few studies have reported until recently. Third, the intervention uses a novel delivery method that provides recommendations at the time of order entry, which is most practical in the ICU environment when the pace of care is at its fastest.

The proposed intervention may result in several benefits considering the impact of delirium on acute and long-term outcomes. It might lead to the development of a universal computerized order entry program that might improve the safety of the current health care system in caring for hospitalized adults with delirium. Our intervention might also decrease health care utilization and cost of the current health care system by reducing days with delirium. Additionally, a successful delirium intervention may also help physicians deliver better care for their patients and increase their trust in the use of information technology as a tool to help their patients.

## Competing interests

The authors declare that they have no competing interests.

## Authors' contributions

All authors have read and approved the manuscript. All authors were involved in study design and manuscript development.

## List of Abreviations

ICU: intensive care unit; GABA: gamma-aminobutyric acid; IRB: institutional review board; WMH: Wishard Memorial Hospital; MICU: medical intensive care unit; SICU: surgical intensive care unit; CCU: coronary intensive care unit; PICU: progressive intensive care unit; RASS: Richmond Agitation-Sedation Scale; CAM-ICU: confusion assessment method for the intensive care unit; CDSS: computerized decision support system; eCHAMP: enhancing care of hospitalized older adults with memory problems; PMD: Pharmacologic Management of Delirium; RMRS: Regenstrief Medical Record System; SAA: serum anticholinergic activity; ACB: anticholinergic cognitive burden; SCCM: Society of Critical Care Medicine; DRS-R-98: Delirium Rating Scale revised 1998; IQCODE: Informant questionnaire on cognitive decline in the elderly; APACHE: acute physiology and chronic health evaluation; SAE: serious adverse event; EPS: extrapyramidal symptom; ANCOVA: analysis of covariance; DSMB: data safety monitoring board; ER: emergency room
